# Predictors of Burden in Carers of Patients with Impulse Control Behaviors in Parkinson's Disease

**DOI:** 10.1002/mdc3.13824

**Published:** 2023-07-26

**Authors:** Daniel Johnson, Leigh Townsend, Anthony S. David, Sally Askey‐Jones, Richard Brown, Mike Samuel, David Okai

**Affiliations:** ^1^ University of Oxford Medical Sciences Division Oxford United Kingdom; ^2^ West Suffolk Hospital NHS Foundation Trust Bury Saint Edmunds United Kingdom; ^3^ Oxford University Hospitals NHS Foundation Trust Oxford United Kingdom; ^4^ Institute of Mental Health University College London London United Kingdom; ^5^ Institute of Psychiatry, Psychology and Neuroscience King's College London London United Kingdom; ^6^ Department of Neurology Kings College Hospital NHS Foundation Trust London United Kingdom; ^7^ Department of Neurology East Kent Hospitals University NHS Foundation Trust Canterbury United Kingdom; ^8^ South London and Maudsley NHS Foundation Trust London United Kingdom

**Keywords:** Parkinson's disease, impulse control disorders, carer burden

## Abstract

**Background:**

Impulse control behaviors (ICBs) are problematic, reward‐based behaviors, affecting 15% to 35% of patients with Parkinson's disease. Evidence exists of increased carer burden as a result of these behaviors; however, little is known about the variables mediating this effect and their management.

**Objective:**

To identify factors predictive of carer burden in a cohort of patients with Parkinson's disease with ICBs to enable the development of targeted therapeutic interventions for carers.

**Methods:**

Data were collected from 45 patients with clinically significant ICBs and their carers, including levodopa equivalent daily dosage, motor and neuropsychiatric symptoms, cognitive function, and ICB severity. Carer burden was quantified by Zarit Burden Interview (ZBI). Univariate analyses were performed using the Spearman rank correlation. Linear regression was used to create a multivariate model for predicting ZBI.

**Results:**

Univariate analysis identified significant correlations between ZBI and patient total Neuropsychiatric Inventory (NPI) (*r*_s_ = 0.50), 4 NPI subscores (agitation/aggression, *r*_s_ = 0.41; depression/dysphoria, *r*_s_ = 0.47; apathy/indifference, *r*_s_ = 0.49; and irritability/lability, *r*_s_ = 0.38; all *P* < 0.02), and the carer 28‐item General Health Questionnaire (GHQ‐28) (*r*_s_ = 0.52, *P* < 0.0005). Multivariate linear regression retained total NPI and GHQ‐28 scores and were collectively predictive of 36.6% of the variance in the ZBI.

**Conclusions:**

Our study suggests that depressive symptoms and aspects of executive dysfunction (apathy and disinhibition) in the patient are potential drivers of carer burden in patients with ICBs. Such findings suggest the presence of executive difficulties and/or mood disturbance should point the clinician to inquire about burden in the caring role and encourage the carer to seek help for any of their own general health problems, which may compound carer burden.

Parkinson's disease (PD) is a neurodegenerative disorder that causes motor, cognitive, behavioral, and psychiatric symptoms. In particular, psychiatric symptoms are known to contribute to carer burden.^1^, ^2^, ^3^ This burden is often attributed to the restrictions in a carer's life and is formally defined as “the extent to which caregivers perceive caregiving to adversely affect their emotional, social, financial, physical and spiritual functioning.”^4^ Increased carer burden has been associated with not only reduced carer quality of life (QoL)^2^, ^5^ but also reduced patient QoL and may lead to premature institutionalization.^6^

Impulse control behaviors (ICBs) are symptoms that may cause carer burden in PD.^7^ These are reward‐based, problematic behaviors that include pathological gambling, compulsive buying, compulsive eating, altered sexual behavior, punding, hobbyism, and dopaminergic medication overuse (dopamine dysregulation syndrome).^8^, ^9^, ^10^ They occur in 15% to 35% of people with PD^11^, ^12^ and are associated with the use of the dopaminergic medication used to treat motor symptoms. ICBs are also associated with high rates of other neuropsychiatric comorbidities, including depression and anxiety.^13^, ^14^ When sufficiently severe as to impact on social and/or occupational function, they are then said to meet “caseness” for the term “impulse control disorder” (ICD), where *caseness* is defined as sufficient severity and range of symptoms to qualify for disorder, and *disorder* is defined as sufficient impact on social and or occupation function to warrant therapeutic intervention. Thus, although the term ICBs may be used to describe the symptoms of an ICD, it also includes the less severe (or subsyndromal) forms of the condition alongside a range of associated behaviors and including repetitive complex (hobbyism) or simple (punding) behaviors or the compulsion to take more medication than prescribed to alleviate dysphoria or achieve a “high.”^9^

Evidence‐based treatments for PD‐ICD are lacking. Structured cognitive behavioral therapy (CBT) has been shown to significantly improve symptoms,^14^ but availability is limited, and medication changes (reduction or cessation) may be required to mitigate symptoms.^15^ Although carers are understood to experience a significant burden in PD‐ICD,^7^, ^14^ their support and engagement are essential in managing PD‐ICD. Indeed, where carer and patient disagree on the presence of an ICD, carer burden is known to be increased compared with cases where they concur.^16^ The carer aids the clinician in identifying and confirming the presence, nature, and severity of ICBs; frequently participates in psychosocial interventions; and encourages patient adherence to ICD treatment programs. The identification of factors associated with carer burden in this cohort may therefore allow for targeted therapeutic intervention for the benefit of carers and therefore patients, optimizing engagement with current treatment options for ICD management.

This study aimed to investigate the factors predictive of carer burden within a cohort of patients with PD with ICBs. We hypothesize that (1) greater severity of ICBs will be positively correlated with carer burden, (2) carers will suffer more burden when patients experience neuropsychiatric symptoms, (3) carer symptoms of physical and mental ill health will increase the risk of carer duress, and (4) motor symptoms will be less of a determining factor in carer burden.

## Patients and Methods

### Study Design, Registration, and Consent

Data analyzed and reported in the present work was collected as part of a randomized, controlled trial (RCT) assessing the benefit of CBT for ICDs.^14^ The RCT was approved by the National Research Ethical Committee (reference no. 08/H0807/1). Separate written informed consent for treatment was obtained from the patient and carer. This trial is registered with isrctn.org (ISRCTN 82636004). Data analyzed for the present work are cross‐sectional and were obtained at the baseline visit.

### Participants and Caregivers

A total of 45 hospital outpatients with their respective carers were recruited for this RCT, and the analysis of the baseline data collected for these participants is described. The research was based at the Institute of Psychiatry, Psychology and Neuroscience, King's College London, with patients recruited from King's College Hospital NHS Trust Regional Neurosciences Centre and UCL Institute of Neurology. The inclusion criterion was a diagnosis of idiopathic PD according to UK PD Society Brain Bank criteria^17^ with an ICB that had failed to remit despite standard measures by the treating neurologist, which could include medication review and changes. ICBs were initially screened for using the Questionnaire for Impulse‐Compulsive Behaviors in Parkinson's Disease.^18^ After a positive screening, ICBs were confirmed in a clinical interview making use of *Diagnostic and Statistical Manual of Mental Disorders–Fourth Edition* criteria for pathological gambling, along with other criteria for the ICB in question by a member of the research team.^14^ Our patients therefore included both those reaching caseness for an ICD as well as some with more mild ICBs only. Exclusion criteria were standardized Mini‐Mental State Examination (sMMSE) score < 24,^19^ non‐English speakers, and no identifiable carer. Participants did not receive financial compensation for their participation in this study.

### Procedure

The study methods have been described in full elsewhere.^14^

### Assessments

The following assessments were made of patients by an appropriately qualified member of the research team: (1) disease severity (Hoehn and Yahr [H&Y] scale, Unified Parkinson's Disease Rating Scale [UPDRS], Clinical Global Impression [CGI]), (2) cognitive function (sMMSE), (3) neuropsychiatric symptoms (Neuropsychiatric Inventory [NPI], Parkinson's Impulse‐Control Scale [PICS] for ICB severity), (4) carer burden (self‐assessment using the Zarit Burden Interview [ZBI]), (5) carer somatic and psychiatric symptoms (self‐assessment using the 28‐item General Health Questionnaire [GHQ‐28]), and (6) dopaminergic medication dosage (levodopa equivalent daily dose [LEDD] was calculated considering both levodopa and dopamine agonist dosages).^20^

The H&Y scale^21^ classifies patients with PD into five stages based on clinical observations of the severity of motor symptoms. The UPDRS^22^ expands on this, assessing patients in the following four domains: nonmotor experiences of daily living, motor experiences of daily living, motor complications, and motor examination. The first three domains were assessed through a structured interview with the patient. The final domain was assessed using a structured clinical examination. The revised 2007 version (Movement Disorder Society–sponsored UPDRS) was used.^23^ CGI^24^ represents the clinician's assessment of overall severity of disease, rated from 1 (normal) to 7 (extremely ill).

The sMMSE^19^ is a standardized and widely used assessment of a patient's overall cognitive function and is rated on a scale from 0 to 30.

The NPI is a semistructured interview with the patient's carer, with the patient in attendance. It assesses the patient's neuropsychiatric symptoms across the following 12 subdomains: delusions, hallucinations, agitations/aggression, dysphoria, anxiety, euphoria, apathy, disinhibition, irritability/lability, aberrant motor activity, appetite and eating abnormalities, and sleep disturbance. The severity and frequency of each symptom is assessed and compiled to give a total NPI score and subscores for each domain.^25^, ^26^

The PICS is a PD‐validated, clinician‐rated scale based on a structured interview with the patient and designed to measure the frequency and impact of the full range of ICBs: gambling, shopping, eating, hypersexuality, simple (punding) or complex (hobbyism) repetitive behaviors, and compulsive overuse of medications. A multiplicative score is calculated for each domain from 0 to 12, which are combined to produce an overall score from 0 to 72.^27^

The ZBI^4^ is a self‐reported measure in which aspects of carer burden are assessed with subjective burden being rated from “never” (0 points) to “nearly always” present (4 points). The 12‐item version, shown to have comparable reliability with the full 22‐item version, was chosen on the grounds of brevity.^28^

The GHQ‐28^29^ is a self‐administered screening tool to detect individuals who have, or are at risk of developing, psychiatric disorders. It comprises subscale domains of depression, anxiety, somatic symptoms, and social withdrawal. We chose the most widely used 28‐item version.

### Statistical Analysis

All analysis was performed using SPSS version 25 (IBM Corp., Armonk, NY). Variables were characterized using the mean and standard deviation. Univariate analyses were performed using the Spearman rank correlation coefficient. Variables showing significant associations were considered for multivariate analyses. Total NPI score was excluded because of the collinearity with the NPI subscores. Backward stepwise linear regression was performed to create a final multivariate model. Collinearity between independent variables was investigated using tolerance scores calculated by collinearity diagnostic options for multiple regression in SPSS. Comparisons between subgroups were performed using *t*‐tests or 1‐way analysis of variance (ANOVA) as appropriate. All statistical analyses were 2‐tailed tests carried out at the 5% significance level.

## Results

### Descriptive Statistics

Data were collected on 45 patients. The mean age (standard deviation) of patients was 58.8 (8.6) years. Of the patients, 68.9% were male (n = 31). The mean duration of PD was 10 (6.1) years, whereas the mean duration of ICBs was 4.2 (3.8) years. All patients had cognition scores within normal limits as assessed by the sMMSE, with a mean of 28.7 (1.4) and range of 24 to 30. Carer burden, as assessed by the ZBI, ranged from 0 to 44, with a mean of 20.7 (10.1). Higher scores indicate greater degrees of burden. Further information regarding patient characteristics is presented in Table 1.

**TABLE 1:** Descriptive statistics of patient group (N = 45)

Patient and Carer Characteristics	Mean (Standard Deviation) or n (%)
Age, y	58.8 (8.6)
Male sex	31 (68.9%)
Years with PD	10.0 (6.1)
Years with ICD	4.2 (3.8)
Medication, on dopamine agonist	24 (53.3%)
Hoehn and Yahr scale stage	2.1 (1.2)
UPDRS I	17.8 (8.0)
UPDRS II	18.1 (10.3)
UPDRS III	29.4 (14.4)
UPDRS IV	8.0 (4.4)
sMMSE	28.7 (1.4)
NPI–total score	24.6 (16.6)
Delusions	0.4 (1.4)
Hallucinations	0.5 (1.4)
Agitation/aggression	2.4 (2.4)
Depression/dysphoria	2.9 (3.4)
Anxiety	4.1 (4.5)
Elation/euphoria	0.4 (1.4)
Apathy/indifference	3.4 (4.3)
Disinhibition	1.8 (2.9)
Irritability/lability	2.8 (2.9)
Aberrant motor behavior	1.2 (3.1)
Sleep and night‐time behavior disorder	3.1 (3.9)
Appetite and eating disorder	2.5 (3.6)
ICBs	
Gambling	16 (35.6%)
Hypersexuality	20 (44.4%)
Shopping	20 (44.4%)
Eating	23 (51.1%)
Hobbyism	26 (57.8%)
Punding	12 (26.6%)
Dopamine dysregulation syndrome	12 (26.7%)
1 ICB	6 (13.3%)
2 ICBs	8 (17.8%)
>2 ICBs	31 (68.9%)
PICS	8.7 (5.4)
LEDD	948.7 (570.5)
Carer–spouse	30 (55.6%)
Carer–son/daughter	7 (15.6%)
Carer–friend/sibling	8 (17.8%)
Carer GHQ‐28–total score	5.5 (6.3)
Zarit Burden Interview	20.7 (10.1)
Minimal/mild burden (≤10)	8 (17.8%)
Moderate (10–20)	12 (26.7%)
Severe (>20)	25 (45.5%)

*Note*: Descriptive statistics of the patient sample are listed.

Abbreviations: PD, Parkinson's disease; ICD, impulse control disorder; UPDRS, Unified Parkinson's Disease Rating Scale; sMMSE, Standardized Mini‐Mental State Examination; NPI, Neuropsychiatric Index; ICB, impulse control behavior; PICS, Parkinson's Impulse‐Control Scale; LEDD, levodopa equivalent daily dose; GHQ‐28, 28‐item General Healthcare Questionnaire.

### Univariate Analysis

The full results are presented in Table 2. Significant correlations with ZBI were observed for total NPI (*r*_s_ = 0.50, *P* < 0.0005) as well as for four of the NPI subscores: agitation/aggression (*r*_s_ = 0.41, *P* = 0.005), depression/dysphoria (*r*_s_ = 0.47, *P* = 0.001), apathy/indifference (*r*_s_ = 0.49, *P* = 0.001), and irritability/lability (*r*_s_ = 0.38, *P* = 0.01). Significant correlations were not observed for other subscores. The carer GHQ‐28 score was also significantly correlated with ZBI (*r*_s_ = 0.52, *P* < 0.0005). Of note, no significant correlation was observed between ZBI and PD severity as assessed by UPDRS subscores, LEDD, or demographic variables. No significant association was identified between carer burden and ICB severity as assessed by the PICS.

**TABLE 2:** Correlations between Zarit Burden Interview score and patient and carer variables

Variable	Correlation Coefficient (*r*_s_)	Significance
Age	0.132	0.387
Years with PD	−0.048	0.752
Years with ICB	0.029	0.861
Hoehn and Yahr scale stage	0.149	0.133
UPDRS I	0.238	0.720
UPDRS II	0.058	0.255
UPDRS III	0.173	0.384
UPDRS IV	0.140	0.249
NPI–total score	0.500**	<0.0005
Delusions	0.103	0.501
Hallucinations	0.151	0.323
Agitation/aggression	0.412**	0.005
Depression/dysphoria	0.468**	0.001
Anxiety	0.174	0.254
Elation/euphoria	0.244	0.106
Apathy/indifference	0.490**	0.001
Disinhibition	0.178	0.243
Irritability/lability	0.379*	0.010
Aberrant motor behavior	0.177	0.245
Sleep and night‐time behavior disorder	−0.21	0.889
Appetite and eating disorder	0.87	0.572
PICS	0.054	0.764
LEDD	−0.177	0.245
Carer GHQ‐28–total score	0.517**	<0.0005

*Note*: Correlations with Zarit Burden Interview using the Spearman rank correlation coefficient.

Abbreviations: PD, Parkinson's disease; ICB, impulse control behavior; UPDRS, Unified Parkinson's Disease Rating Scale; NPI, Neuropsychiatric Index; PICS, Parkinson's Impulse‐Control Scale; LEDD, levodopa equivalent daily dose; GHQ‐28, 28‐item General Healthcare Questionnaire.

* Correlation coefficient *P* < 0.05.

** Correlation coefficient *P* < 0.01.

Further analysis was performed to examine the relationship between different ICBs, measured by subscores of the PICS, and ZBI. No significant between‐group differences were seen for patients with different ICBs (*P* = 0.81) using 1‐way ANOVA. In addition, no significant correlations were found between individual domain PICS subscores (eg, gambling) and ZBI (although the shopping subscore approached significance: *r*_s_ = 0.62, *P* = 0.055). No significant difference was seen (*P* = 0.55) when comparing participants with a PICS subscore >4 (likely ICD)^27^ (n = 26) and those without (n = 19).

### Multivariate Analysis

To determine the variables best predicting carer burden, carer GHQ‐28 score, total NPI score, and NPI subscores were entered into a backward multiple linear regression model. ZBI was used as the dependent variable. The total NPI and carer GHQ‐28 scores were retained in the optimal model and were found to account for 36.6% of the variance in ZBI. Data are presented in Tables 3 and 4. To investigate possible collinearity between total NPI and its subscores, variable tolerances were tested. This was not indicative of problematic collinearity, with all independent variables having a tolerance of at least 0.341.

**TABLE 3:** Results from multivariate linear regression analysis

Model	Retained Variables	B	Standard Error	β	Significance
1	NPI–total score	0.127	0.127	0.212	0.323
	Carer GHQ‐28–total score	0.612	0.253	0.383	0.021
	NPI apathy/indifference	0.395	0.394	0.167	0.323
	NPI depression/dysphoria	0.363	0.495	0.123	0.468
	NPI irritability/lability	−0.28	0.614	−0.81	0.651
2	NPI–total score	0.096	0.106	0.161	0.37
	Carer GHQ‐28–total score	0.565	0.229	0.353	0.018
	NPI apathy/indifference	0.452	0.369	0.191	0.229
	NPI depression/dysphoria	0.364	0.490	0.129	0.463
3	NPI–total score	0.136	0.091	0.228	0.141
	Carer GHQ‐28–total score	0.595	0.224	0.372	0.011
	NPI apathy/indifference	0.476	0.366	0.201	0.201
4	NPI–total score	0.191	0.081	0.319	0.023
	Carer GHQ‐28–total score	0.679	0.216	0.425	0.003

*Note*: Results from multivariate linear regression after each step of backward regression analysis. Initially entered variables: NPI, agitation/aggression; NPI, depression/dysphoria; NPI, apathy/indifference; NPI, irritability/lability; NPI–total score; carer GHQ‐28–total score.

Abbreviations: NPI, Neuropsychiatric Index; GHQ‐28, 28‐item General Healthcare Questionnaire.

**TABLE 4:** Summary statistics of models used in multivariate regression

Model	*R*	*R* ^2^	Adjusted *R* ^2^	Standard Error of the Estimate
1	0.658	0.433	0.356	8.131
2	0.655	0.430	0.370	8.045
3	0.649	0.421	0.377	7.999
4	0.630	0.396	0.366	8.068

*Note*: Summary statistics are listed for each of the models used in the multivariate regression analysis. Variables included in each model are listed in Table 3.

## Discussion

Our study, in a large cohort of patients with PD‐ICB, is the first to adopt an inclusive dimensional approach to investigate the degree of carer burden in relation to the presence of ICBs as we used the PICS, a rating tool validated in PD‐ICD, rather than the more usual categorical (ICD present or absent) approach.^7^, ^27^ In our cohort, more than half of carers experienced carer burden to some degree. The severity of burden perceived appears broadly in line with other studies that have made use of the ZBI administered to carers of patients with PD.^30^, ^31^ Total neuropsychiatric burden and the carer's own health appeared to be the greatest predictors and showed a modest but significant degree of influence on carer burden. Conversely, we found no significant association between ZBI and ICB severity, as assessed by the PICS.

Our finding of the importance of patients' neuropsychiatric symptoms in influencing carer burden is consistent with the literature in PD and in PD‐ICBs, where depression and apathy in particular have previously been highlighted as a relevant risk factors.^1^, ^5^, ^7^ Although the total neuropsychiatric load appears to be the main driver of burden, it is also of interest that low mood, apathy, and social disinhibition in particular appear to be the greater of the subcategory factors most correlated with such an outcome. This again is most consistent with the literature in patients with PD, PD dementia, and PD‐ICB.^32^ The former of these 2 factors in particular have also been found in other studies to be associated with the presence of ICBs.^12^, ^33^

In addition to the lack of an association between ICB severity and carer burden, we did not find associations between ZBI and PD or ICB duration, overall disease progression (H&Y stage, UPRDS domains), or LEDD. The lack of association with disease stage, given its ubiquity in the wider PD literature,^34^ is possibly an artifact of our relatively small sample. However, it may also be a consequence of the relative enrichment, in our PD‐ICD cohort, for a more prominent and burdensome neuropsychiatric phenotype, irrespective of disease stage. Regarding LEDD, although an association has been reported between this and carer burden,^35^ the absence of such an association in our work may reflect the observation that while in patients with PD without ICDs, being *off* dopaminergic medication moderates heightened reward sensitivity, in PD‐ICD, this sensitivity remains regardless of the presence or absence of medication.^33^

In our study, we observed the same high levels of carer burden as measured by ZBI seen in previous studies of patients with PD‐ICD with around 50% of carers experiencing a comparable,^28^, ^36^ severe degree of burden.^7^, ^32^ However, the lack of a direct correlation between ICB severity and carer burden is of interest given their often highly damaging consequences for the patient–carer relationship clinically.^37^ We investigated whether different ICBs might have differing associations with carer burden but found no significant differences in ZBI between patients with different ICBs nor any significant correlations between ZBI and PICS subscores. This may be attributed to the significant comorbidity between different ICBs seen in our cohort, where 70% of patients displayed >2 ICBs. There may also be a selection bias in that our cohort was recruited from patients with refractory ICBs, which may have somewhat standardized ICB severity. Such subgroup analyses are also limited by a reduced sample size and consequently low power.

Neither a large‐scale cross‐sectional study (n = 633) from neurology clinics, addressing some compulsive behaviors (buying, gambling, and hypersexuality),^38^ nor others comparing those with ICBs/ICD^7^, ^32^ found a direct association with ICBs and carer burden, but both found depression and apathy to be mediators of burden. Finally, in the present cohort, assessment after structured CBT resulted in significant improvements in severity and frequency of ICBs at the conclusion of treatment,^14^ but did not result in an improvement in carer burden. Collectively, these results suggest that the association of ICBs with increased carer burden may be a secondary consequence of the increased neuropsychiatric symptom burden present in these patients.

A potential unifying factor explaining the relationship between neuropsychiatric symptoms, ICBs, and carer burden may be executive dysfunction. Our observed findings demonstrate that apathy, aggression, depression, and irritability occur commonly in a PD‐ICD cohort and that these features, as indexed by the NPI, are significantly correlated with greater carer burden. In the International Classification of Diseases, Tenth Revision, the clinical manifestation of this executive dysfunction, referred to as an “organic personality disorder,” includes both aspects of apathy and disinhibition in its subclassification, referred to respectively as “pseudo‐depression”^39^ and “pseudo‐psychopathy.” The 2 clinical phenotypes overlap in a striking manner in our current observations, although we also note that important clinical differential diagnoses would include personality changes results from medication use, secondary impulse control, or addictive behavior syndromes (International Classification of Diseases, Eleventh Revision). Unfortunately, our study lacked the range of tools to investigate executive impairment and its relationship to behavior problems.^40^, ^41^, ^42^, ^43^ Where such behavioral phenotypes have been more explicitly evaluated, for instance, in studies comparing carer distress in behavioral variant frontotemporal dementia (bvFTD) and Alzheimer's disease,^44^ findings indicate distress to be significantly higher in the bvFTD group than the more cortical (cognitive)‐based Alzheimer's disease dementia. Executive function has also been seen to be a strong independent predictor of carer burden in a cohort of patients with early‐stage PD, although the presence of ICBs was not assessed.^45^ This may explain the CBT RCT by Okai et al.,^14^ in which observed improvements in symptoms, including depression and ICB severity, did not result in a marked improvement in carer burden.

Finally, we also found the global rating of carer health to be related to carer burden. There was no difference in the various subdomains (depression, anxiety, somatic symptoms, and social withdrawal) of the GHQ‐28, although the study was likely underpowered to detect such a difference. These findings are similar to the findings in PD more generally, where QoL markers that include physical and mental health well‐being were found to correlate with carer burden.^23^, ^46^

There are some limitations to this work, which should be considered. First, the study was drawn from a population of patients who were either unable to tolerate dopamine agonist withdrawal or whose ICBs had persisted despite the withdrawal, potentially limiting generalizability, as patients in this study may have had more resistant ICDs compared with patients with PD in whom ICDs resolved on drug manipulation. Second, the cross‐sectional nature of the work means that it is not possible to establish causality; it may be that carers who are already under strain or are experiencing poorer health will report greater levels of irritability, aggression, and loss of empathy in those they cared for than were actually present. Similarly, although carer‐assessed NPI is a commonly used tool for the assessment of neuropsychiatric symptoms in PD, it is conceivable that poor carer health, both physical and mental, and the presence of burden in of itself, may bias reporting of neuropsychiatric symptoms. Finally, carer roles and relationships were varied and complex and were not fully explored in the current study design. Although, as is standard for most psychiatric assessments, efforts were made to assess patients jointly with carers present to ensure consistency of view, the potential effects of such variation in patient–carer relationships are beyond the scope of this report but should be considered an important topic for future work. We propose that future work should aim to test our findings, examining the relationship between ICBs, neuropsychiatric symptoms, and executive function in a longitudinal sample of patients with PD‐ICD and their carers. This work should use clinician, carer, and patient rated scales to minimize the impact of varied confounders, and the collection of data on carer physical and mental health would allow investigators to account for these factors in their analysis. The inclusion of patients with PD with resolved ICBs, or drawn from a general PD population, without ICBs would have enabled greater certainty in our conclusions and potentially widened their application, therefore future work should aim to include a comparator cohort when further evaluating the relationship between carer burden and ICBs.

In summary, factors associated with an increase in carer burden include neuropsychiatric aspects of the disease itself and aspects linked to the carer. ICB severity does not appear to be a significant predictor of carer burden. This study highlights the need for a thorough clinical evaluation in patients with ICBs to identify other neuropsychiatric symptoms and factors affecting the health of the carer, as these appear to be combined risk factors impacting on the patient–carer dyad. Subsequently, it is likely that any management of ICBs will need to be in the context of reducing overall neuropsychiatric burden in the patient and addressing the quality of social support and coping strategies available for carers.

## Author Roles

(1) Research Project: A. Conception, B. Organization, C. Execution; (2) Statistical Analysis: A. Design, B. Execution, C. Review and Critique; (3) Manuscript Preparation: A. Writing of the First Draft, B. Review and Critique.

D.J.: 2A, 2B, 3A, 3B

L.T.: 3A, 3B

A.S.D.: 1A, 3B

S.A.‐J.: 1B, 1C

R.B.: 1A, 3B

M.S.: 1A, 3B

D.O.: 1A, 1B, 1C, 2A, 2C, 3B

## Disclosures

**Ethical Compliance Statement:** The study was approved by the UK National Research Ethics Committee (reference no. 08/H0807/1). Separate informed written consent was obtained from patients and carers. We confirm we have read the Journal's position on issues involved in ethical publication and we affirm that this work is consistent with those guidelines.

**Funding Sources and Conflicts of Interest:** The study reported in this article was supported by grant support (A.S.D., R.B.) from Parkinson's UK (reference J‐0705). This study represents independent research part‐funded by the National Institute for Health Research (NIHR) Maudsley Biomedical Research Centre at South London and Maudsley NHS Foundation Trust and King's College London and the NIHR Biomedical Research Centre, University College Hospital. The views expressed are those of the author(s) and not necessarily those of the NHS, the NIHR, or the Department of Health and Social Care.

**Financial Disclosures in the Previous 12 Months:** A.S.D. received research funding from the NIHR UK, Parkinson's UK, and the Medical Research Council, UK. R.B. received funding from the NIHR, part salary support through the Biomedical Research Centre for Mental Health, South London, and Maudsley NHS Foundation Trust. M.S. received honoraria for consultancy from Abbott, and King's College Hospital receives support for educational meetings from Parkinson's UK. D.J., L.T., S.A.‐J., and D.O. have nothing to disclose.
